# Precursor or Sequela: Pathological Disorders in People with Internet Addiction Disorder

**DOI:** 10.1371/journal.pone.0014703

**Published:** 2011-02-16

**Authors:** Guangheng Dong, Qilin Lu, Hui Zhou, Xuan Zhao

**Affiliations:** 1 Department of Psychology, Zhejiang Normal University, Jinhua, People's Republic of China; 2 Institute of Neuroinformatics, Dalian University of Technology, Dalian, People's Republic of China; RAND Corporation, United States of America

## Abstract

**Background:**

This study aimed to evaluate the roles of pathological disorders in Internet addiction disorder and identify the pathological problems in IAD, as well as explore the mental status of Internet addicts prior to addiction, including the pathological traits that may trigger Internet addiction disorder.

**Methods and Findings:**

59 students were measured by Symptom CheckList-90 before and after they became addicted to the Internet. A comparison of collected data from Symptom Checklist-90 before Internet addiction and the data collected after Internet addiction illustrated the roles of pathological disorders among people with Internet addiction disorder. The obsessive-compulsive dimension was found abnormal before they became addicted to the Internet. After their addiction, significantly higher scores were observed for dimensions on depression, anxiety, hostility, interpersonal sensitivity, and psychoticism, suggesting that these were outcomes of Internet addiction disorder. Dimensions on somatisation, paranoid ideation, and phobic anxiety did not change during the study period, signifying that these dimensions are not related to Internet addiction disorder.

**Conclusions:**

We can not find a solid pathological predictor for Internet addiction disorder. Internet addiction disorder may bring some pathological problems to the addicts in some ways.

## Introduction

Internet use has increased considerably over the last decade. Data from China Internet Network Information Center (CNNIC) as of June 30, 2010 showed that 420 million people go online, 58.0% of whom range between 10-29 years old [1]. The soaring number of Internet users have resulted in an increased population percentage being afflicted with the medium's problematic use, now referred to as Internet addiction disorder (IAD). IAD has become a serious mental health problem not only in China, it appears to be a common disorder that manifested worldwide and merits inclusion in DSM-V [2], [3]. In Germany, 9.3% reported at least one negative consequence of Internet use, especially neglect of recreational activities and problems with family/partner, work or education, and health [4]. Chou and Hsiao reported that the incidence rate of Internet addiction among Taiwanese college students is 5.9% [5]. In addition, Wu and Zhu reported that 10.6% of Chinese college students suffer from Internet addiction [6]. South Korea considers Internet addiction one of its most serious public health issues [2].

Understanding IAD is important because of its association with other psychiatric illnesses, such as pathological and compulsive behaviors [7]. It has been reported that extensive Internet use may bring forth a heightened level of psychological arousal [8], possibly resulting in online users experiencing health problems [9], [10]. Several studies claim the underlying psychopathology of Internet addiction, including depression, social anxiety, and substance dependence [11], [12]. Although methodological problems have hindered the full power of these studies [13]. IAD subjects (hereinafter referred as IADs) usually manifest abnormal behaviors, such as anxiety, depression, or isolation. However, it either is not clear if these factors are precursors of IAD or sequela from IAD. In fact, IAD researchers currently face this controversial issue.

From a clinical psychiatry perspective, a profile of Internet addicts may include individuals who have one or more of the following dimensions: depression, bipolar disorder, sexual compulsion, and loneliness. Morahan-Martin argued that it is difficult to determine causality between the pathological dimensions and the IAD, and that Internet addiction may be symptomatic of other disorders (e.g., pathological behavior) [14]. The cognitive-behavioral model on IAD suggests that psychopathology is a distal necessary cause of IAD symptoms (i.e., psychopathology must be present or must have occurred for the symptoms of IAD to occur) [15]. Armstrong et al. studied impulsivity and self-esteem as measures of addiction, showed that self-esteem was a better, but not an absolute predictor, of Internet addiction [16],. Thatcher and Goolam argued that high risk groups associate their time allocated online with excitement and independence [17].

Internet allows an individual to unlock his or her personality and create a persona that may be very different from reality [10], [18]. The appeal of the medium can be attributed to the fact that real-life constraints can be isolated, and that experimentation with altered perceptions is possible (e.g., construction of the ideal self). Individuals with lower self-esteem have been associated with increased hours of Internet usage, perhaps as a form of escape. Shapira et al. believe that IAD is “an individual's inability to control Internet use, which in turn leads to feelings of distress and functional impairment of daily activities” [7].

All of these studies provide valuable information in understanding the characteristics of IAD. They have investigated the current state of mind of people suffering from the said addiction disorder. However, it is difficult to determine the causality between pathological problems and IAD. For example, which of these factors is precursor for addiction or result from addiction? In one hand, people manifesting a certain level of pathological problem were known to be easily addicted to the Internet. On the other hand, IAD may change an individual's mental status, and consequently, bring forth some type of pathological disorder. Horizontal studies can not explain this dilemma clearly. Thus, a longitude study was conducted in order to identify the causal relation.

In the present study, we used longitude research methods to identify the pathological problems in IAD, as well as to explore the mental status of IAD prior addiction, including the pathological traits that may trigger IAD. Data from Symptom Checklist-90 (SCL-90) was obtained from 59 subjects before and after their suffering from IAD. It is believed that comparisons of data before IAD, the norm usage of Chinese people, and data collected after IAD may bring useful information on this topic.

## Methods

### Symptom Checklist SCL-90

The SCL-90 [19] is an instrument for the measurement of psychological distress and certain aspects of psychopathology. It comprises 90 statements that describe physical and psychic symptoms. Subjects were asked to indicate the amount they were bothered by each of the symptoms over the past week on a 5-point Likert scale ranging from “not at all” (0) to “extremely” (4). Applying factor analysis, Derogatis [19] had derived nine subscales or dimensions from the instrument which he labeled somatisation (SOM), obsessive-compulsive (O-C), interpersonal sensitivity (INT), depression (DEP), anxiety (ANX), hostility (HOS), phobic anxiety (PHOB), paranoid ideation (PAR), psychoticism (PSY), and additional items (ADD). A high score in a given dimension indicates high expression of the corresponding distress. The Chinese version of SCL-90, as it was adapted and tested by Wang [20] and were widely used in researches and clinical measures in China [21].

### Young's Online Internet Addiction Test

Young's online Internet addiction test has 20 items associated with online Internet use, including psychological dependence, compulsive use, and withdrawal, as well as the related problems of school or work, sleep, family, and time management. For each item, a graded response is selected from 1 =  “Rarely” to 5 =  “Always”, or “Does not Apply”. People scored more than 50 were thought experiencing occasional or frequent problems because of the Internet. People scored more than 80 was thought causing significant problems in their life [22]. In present study, participants scored more than 80 were viewed as Internet addicts.

### Participant Selection

In September 2008, 2132 freshman students were tested using the SCL-90. The data were obtained from 1024 (48%) female and 1108 (52%) male student. In Sept. 2009, all of them were tested by Young's online Internet addiction test. To control participants' exposure time to the Internet, students majoring in software, computer information, and related fields were excluded from survey. By Young's definition [9], a total of 66 students (12 female) were judged to be Internet addicts in this study. In order to know if these 66 students were addicted to Internet when they just entered the university (Sept. 2008), a retrospective diagnosis was assessed on these Internet addicts. Seven addicted male students were excluded because their classmates or tutors reported that they were familiar with the Internet when they entered the university. This is to guarantee that all changes were taken placed in their first year in the subjects. The other 59 students were unfamiliar with the Internet as freshmen; however, one year later, they were diagnosed as addicted to the Internet. Also, the mental states of these 59 IADs were measured using SCL-90 (Sept. 2009). The first test of SCL-90 was arranged by the university (It is the university's policy to know all students' mental fitness when they entered the university). So, no informed consent forms were signed. In the second time, each subject signed an informed consent form for the study. The research procedure was in accordance with the ethical principle of the 1964 Declaration of Helsinki (World Medical Organization). The institute review board of Zhejiang Normal University approved the research procedure.

## Results

**Figure 1:**
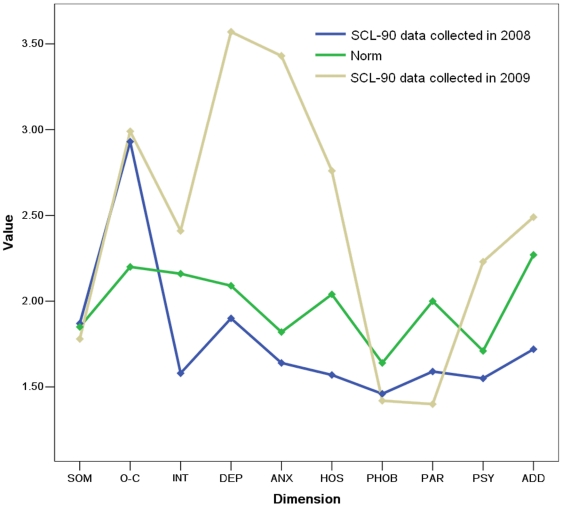
Mean scores of SCL-90 dimensions in different groups. The figure shows the characteristics of different dimensions in different measures. From this figure, we can see that INT, DEP, ANX, HOS and PSY changed fiercely between data collected in 2008 and 2009. However, SOM, O-C, and PHOB showed little changes.

Single-sample *t* tests were conducted among 59 Internet addicts and the norm of Chinese people. Next, paired-samples *t* test were conducted between the SCL-90 data collected in 2008 and 2009 from these 59 students. Table 1 shows the means and standard deviations of SCL-90 data collected in 2008 and 2009, and the norm values for Chinese people. The characteristics of each dimension are shown in Figure 1.

**Table 1:** Mean scores of SCL-90 dimensions in different groups.

Dimension	SCL-90 data 2008 (n = 59)		Norm of Chinese People	SCL-90 data 2009 (n = 59)	
	*M*	*SD*		*M*	*SD*
SOM	1.87	0.47	1.85	1.78	0.50
O-C	2.93	0.56	2.2	2.99	0.52
INT	1.58	0.30	2.16	2.41	0.79
DEP	1.90	0.43	2.09	3.57	0.44
ANX	1.64	0.46	1.82	3.43	0.60
HOS	1.57	0.47	2.04	2.76	0.70
PHOB	1.46	0.58	1.64	1.42	0.49
PAR	1.59	0.47	2	1.64	0.57
PSY	1.55	0.51	1.71	2.23	0.62
ADD	1.72	0.53	2.27	2.29	0.70

*Notes*: *M*, arithmetic mean; *SD*, standard deviation.

Upon comparison, only O-C in SCL-90 results (2008) showed a significantly higher score compared to the norm (Table 2). Significant differences were found in O-C, DEP, ANX, and HOS dimensions when SCL-90 results (2009) and the norm were compared. Results in SCL-90 (2009) showed significant and increasing scores for INT, DEP, ANX, HOS, and PSY, as compared to the results in SCL-90 (2008) (Table 2).

**Table 2:** Comparison results among different types of data.

Dimension	SCL-90 data (2008) minus norm		SCL-90 data (2009) minus norm		SCL-90 data (2009 minus 2008)	
	Difference	*p*	Difference	*p*	Difference	*p*
SOM	0.02	0.737	−0.07	0.278	−0.09	0.314
O-C	0.73	0.000	0.79	0.000	0.06	0.585
INT	−0.58	0.000	0.25	0.017	0.83	0.000
DEP	−0.19	0.001	1.48	0.000	1.67	0.000
ANX	−0.18	0.003	1.61	0.000	1.79	0.000
HOS	−0.47	0.000	0.72	0.000	1.19	0.000
PHOB	−0.18	0.021	−0.22	0.000	−0.04	0.633
PAR	−0.41	0.000	−0.36	0.000	0.05	0.552
PSY	−0.16	0.024	0.52	0.000	0.68	0.000
ADD	−0.55	0.000	0.02	0.855	0.57	0.000

*Notes:* In SCL-90 results, if people scored lower than the norm, they were thought healthy in this dimension.

## Discussion

### Mental States before Addiction

Based on the comparison, we find that the scores of the 59 students were lower than the norm for most of the SCL-90 dimensions before their addiction. Only the score of O-C (obsessive-compulsive) dimension among IADs was significantly higher than Norm. The result suggests that people showed more O-C behaviors before they became addicted to the Internet. In fact, addiction is usually defined as a brain disease that manifests as a compulsive behavior, or the compulsive and continued use of a substance or behavior even if the user considers it harmful [23]. This result is consistent with Shapria's study that IADs usually manifest compulsive behaviors [7]. Studies on individuals suffering from substance [24] and tobacco [25] addictions also showed evident in O-C behaviors. Therefore, the relation between O-C and IAD was easily confirmed.

### When People Become Addicted to the Internet

The current mental states of IADs can be explored by comparing IAD09 and the norm. Results show that the scores of O-C, DEP, ANX, and HOS in IADs were significantly higher than norm, suggesting that students suffering from IAD likewise currently suffer from the above-mentioned pathological problems. For SOM, INT, PHOB, PAR, PSY, and ADD, findings suggest that IAD is not related with these dimensions. Meanwhile, depression and anxiety were proven types pathological problems associated with IAD in previous studies [14], [16]. The present study therefore supports related findings on DEP and ANX. Previous studies have likewise found that hostility is associated with Internet addiction among males [26]. Hostility has been reported to predict escape-avoidance coping styles, as well as substance use triggered by known cues (e.g., negative emotional states and tension) [27]. For adolescents, higher hostility usually leads to interpersonal conflict and rejection. Since substances are made less available to them, the Internet could provide a virtual world to escape from stress from the real world [28].

### Highlights on the SCL-90 Results from 2008 and 2009

The comparative results between the data collected in 2008 and 2009 provide the mental states in these 59 Internet addicts that changed during the year. The scores for INT, DEP, ANX, HOS, and PSY changed significantly during this year. However, scores for SOM, O-C, PHOB, and PAR did not change significantly, suggesting that these dimensions are not related to IAD. Previous studies have in fact shown the harm induced by IAD, such as mood disorders, attentional disorders, and substance dependencies were cited as comorbidities [29], [30]. As such, when comorbid disorders are addressed alongside IAD, patient outcomes can be greatly improved [31].

### Precursor or Sequela

The features of SCL-90 dimensions in present study can be divided into four types. First, SOM, PAR, and PHOB did not change greatly before and after their addiction, which means that these dimensions were neither **precursors nor Sequela** of IAD. Simply put, they showed no relationship with IAD. Second, the O-C score was much higher than the norm before IAD, and thus, could be considered a predictor for IAD. However, The O-C score did not change significantly in 2009, which may somehow affect the reliability of this finding. In one hand, results suggest that the O-C can be a predictor of IAD since it showed a higher score before Internet addiction. Yet, since O-C score did not change significantly in 2009, the O-C dimension may not be related to IAD. As such, we can not absolutely conclude the certainty of O-C being a predictor of IAD.

Third, before their addicted to Internet, the scores of DEP, ANX, and HOS for students with IAD were lower than the norm, which means that nothing erroneous was found in these dimensions. Essentially, these dimensions can not be categorized as predictors of IAD. After their addiction, the dimensions scored high and even increased significantly, suggesting that DEP, ANX, and HOS were outcomes of IAD, and not precursors for IAD. This finding may help us better understand the causality between pathological disorders and IAD [15], [17]. The fourth type, which focuses on INT and PSY, showed that these dimensions were normal before Internet addiction. Although their scores were not significant in relation to the norm compared to the SCL-90 data collected in 2009, it was observed that they changed significantly in 2009, as evidenced by the comparison between the SCL-90 data collected in 2008 and 2009. As such, we can conclude that the increased score for INT and PSY dimensions were outcomes of IAD.

A large number studies have explored the predictors of Internet addiction. Communication pleasure [5], impulsivity [32], and competition and cooperation [33] were proven predictors of Internet addiction. Most of these studies have emphasized on experiences in using the Internet and personality traits as related to Internet addiction. However, only few studies clearly explored its causality with pathological disorders. The results of present study may further our understanding about the relationship between pathological disorders and Internet addiction. Thus, the causal relationship between pathological disorders and Internet addiction should be further evaluated by prospective studies.

### Limitations and Shortcomings

Results from present study revealed several important findings to deepen our understanding about the pathological disorders of Internet addiction, however, several limitations should be considered. First, this research lasted for one year. During this year, many things happened that might change a person's mental states. So, it is hard to make conclusions with 100 percent certainty that these changes were related with IAD. Second, the SCL-90 is a useful tool in measuring the mental states in the recent past week, however, it can not trace the changing process during a longer period. This research only showed the static mental states of the students before and after their addicting to Internet. Third, the number of IADs is limited (59), more participants should be found if possible in future studies. Fourth, we used the norm but not the data from control group as comparison level. This is because it is very hard to do another extensive investigator as the first measure in present study. Using the norm as comparative level is useful and easy.

Although there are so many limitations in this study, we still believe it is valuable. First, it is harder to control extra variables in longitudinal studies than in experimental studies, especially studies with patients. Second, the present study showed that it is hard to find a solid predictor for IAD, which is different from previous study results. It broadened our knowledge about IAD.

### Conclusions

In summary, we can find that there are no solid pathological predictors for IAD. While O-C may be considered as one dimension, it remains that this finding cannot be absolutely concluded. On the contrary, Internet addiction disorder may bring some pathological problems to the people who suffering from it, although, the conclusion still need more support because of the limitation of the research design in present study.
